# Optimization of low-temperature nitrogen plasma in reducing fungi and aflatoxin human exposure through maize

**DOI:** 10.1038/s41598-025-95153-0

**Published:** 2025-04-05

**Authors:** Hannah Mugure Kamano, Michael Wandayi Okoth, Wambui Kogi-Makau, Patrick Wafula Kuloba, Joshua Ombaka Owade, Patrick Murigu Kamau Njage

**Affiliations:** 1 Food Technology Research Centre, Kenya Industrial Research & Development Institute, P.O Box 30650, Nairobi, 00100 Kenya; 2 Department of Food Science & Technology, University of Nairobi, P.O Box 29053, Nairobi, 00100 Kenya; 3 Department of Biosystems and Agricultural Engineering, Michigan State University, Duduville Campus, P.O Box 45917 – 00100, East Lansing, Michigan 48824 USA; 4 Research Group for Genomic Epidemiology, Technical University of Denmark, Lyngby, Denmark

**Keywords:** Low-Temperature nitrogen plasma (LTNP), Aflatoxin, Fungi, Response surface methodology (RSM), Box Behnken design, Maize, Quantitative exposure assessment, Computational models, Fungi

## Abstract

Globally, aflatoxin contamination in maize remains a huge burden despite many interventions put in place. The use of low-temperature plasma to decontaminate the maize is a potential solution for ensuring the safety and extended shelf life of the grain. This study optimized the parameters and investigated the efficacy of low-temperature nitrogen plasma (LTNP) in destroying fungi and reducing exposure to aflatoxins in naturally contaminated maize from an endemic region. The study generated 17 experimental runs using the Response Surface Methodology (RSM) of the Box Behnken Design (BBD) with exposure time, pressure, and ionization density as independent variables. Quantitative exposure assessment was conducted using Monte Carlo simulations followed by sensitivity and scenario analysis to study factors influencing exposure and best aflatoxin-reducing plasma parameters. The best-fitting RSM model, the linear model, indicated that increased exposure time but not pressure and power led to a corresponding statistically significant decrease in the fungal load and aflatoxin content. LTNP reduced aflatoxin contamination to levels below all the main global regulatory limits. Numerical optimization of the percent reduction in aflatoxin and fungal load indicated that an exposure time of 1793.4 s, pressure of 0.98 pascal and ionization power of 189.8 W are required to achieve an optimal reduction of aflatoxin content of 82.6% and fungal load of 96.9%. Exposure assessment indicated high exposure especially for populations with lower body weight with ρ = -0.46 between body weight and exposure. The best LTNP combinations achieved aflatoxin exposure reduction results comparable to but with markedly less variation than existing practically used decontamination methods. Further optimization studies during upscaling are recommended, incorporating independent factors such as temperature and processing volume and outcomes such as organoleptic, physical, and chemical changes in the food matrices after treatment.

## Introduction

Aflatoxins are produced by aflatoxigenic fungi particularly *Aspergillus flavus*, *Aspergillus parasitucus* and more rarely *Aspergillus nomius*^1,2^. Fungi are able to grow on a broad spectrum of foods such as cereals, fruit, vegetables, meat, fats, and many others, which eventually leads to the production of toxins, development of off-flavour, rotting, changes in colour and growth of pathogens^2,3^. The intake of high aflatoxin concentration may lead to liver cirrhosis & death^4,5^. For instance, Kenya has featured several unprecedented epidemics in human history^6–8^. Long-term exposure to low concentrations has, on the other hand, been associated with cancer, stunted growth, stillbirths, jaundice^9^, and immune system suppression^6^. Globally, 25,200–155,000 of new liver cancer cases are attributed to aflatoxins^9^. There are significant economic losses due to aflatoxin contamination in the maize industry which is estimated to lose $52.1 million to $1.68 billion in the United States due to aflatoxin contamination attributable to climate change^10^.

Several approaches for aflatoxin decontamination in food, including physical, chemical, and biological categories, have been investigated. Physical methods, such as sorting, Ultraviolet (UV) light exposure, and heat treatment, help reduce contamination but may not always fully eliminate toxins. Chemical methods, including the use of ammonia and hydrogen peroxide, degrade aflatoxins but introduce the risk of chemical residues in food products and may negatively affect the environment^11,12^. Biological control, using non-toxic strains of Aspergillus or bacteria to inhibit aflatoxin production, is a promising pre- and post-harvest intervention approach^13^.

Low-temperature plasma (LTP), on the other hand, is an emerging technology in the food industry that is capable of rapidly decontaminating a food matrix at ambient temperature and pressure while leaving the food without any detectable changes in quality. Plasma is the fourth state of matter that consists of an ionized gas comprising several reactive species (RS) that include electrons, photons, negative and positive ions, free radicals and molecules^14–17^. These RS facilitate a rapid decontamination process and are selective in nature thereby damaging the pathogen while leaving the host unharmed. This occurs at relatively low temperature and pressure without causing any major changes in the food quality^17,18^. LTP is an affordable and sustainable approach in the long run especially when air is used instead of commonly used gases such as Helium, Argon, Nitrogen, and Heliox^18^. LTP has been investigated in the food industry, where it has shown the potential to replace conventional decontamination methods because of its high efficacy and efficiency^19–22^.

Cold plasma has been found to have more superior results in aflatoxin decontamination and fungal inactivation when compared to traditional approaches^23,24^. The mechanism of inactivation of fungi by LTP is through chemical interaction of the plasma species with the specimen, destruction of the membranes as well as the internal cellular structures and finally breaking of the DNA strands of the fungi^25–27^. Similar to that of fungi, the mechanism of decontamination of mycotoxins using LTP is not exhaustively studied. However, the mycotoxin degradation is associated with their molecular structure and the type of plasma which consequently affects the kind of interaction that results. The free radicals that result from the ionization may be linked to the degradation of the mycotoxins^17,28^. It is of interest that the same oxidative stress mechanism whereby aflatoxin biosynthesis is induced^17,29^ may also be the main mechanism through which LTP reduces aflatoxin production through induction of oxidative stress in aflatoxin-producing fungi and degradation of aflatoxins. Reactive oxygen species play a central role in inducing aflatoxin biosynthesis in response to environmental stresses and aflatoxin production in *A. parasiticus* and *A. flavus *is attributable to their oxidative stress response^29^.

Though LTP is a promising technology in reducing aflatoxin contamination in maize, there are no studies optimizing the application of the technology and its parameters in reducing consumer exposure to aflatoxins in maize. Studies are also needed that study the effectiveness of LTP application in population exposure amelioration through studies on naturally contaminated maize from endemic regions. Whereas laboratory challenge tests on maize provide consistent, controlled aflatoxin distribution, leading to predictable results, naturally contaminated maize offers more realistic conditions, reflecting the irregular and patchy contamination patterns seen in agriculture. The advantage of using naturally contaminated maize is that it mirrors real-world challenges such as uneven contamination, moisture variability, and diverse fungal strains and aflatoxin types (e.g., B1, B2, G1, G2) under varying environmental conditions, making the findings more applicable for large-scale food safety interventions. The degradation of mycotoxins is dependent on their structure and the presence of the matrix; pure toxins are degraded faster than a mixture of several mycotoxins^30^. Probabilistic exposure assessment is a useful approach to take this greater variability in treatment outcomes under natural contamination into account^31^. Shi et al.^32^in their study on the effect of cold plasma on aflatoxin in laboratory challenge tests on maize, reported a 62–82% decrease in aflatoxin content using high-voltage atmospheric cold plasma. They also reported that properties of the type of plasma produced are dependent on several factors: the type of gas used, matrix under treatment, process parameters and equipment type^32^. Wielogorska et al.^33^on the other hand, reported an aflatoxin reduction of up to 66% in maize using cold atmospheric pressure plasma. Optimization experiments often follow the one-factor-at-a-time technique^34^. This requires volumes of data to identify the optimum level, is time-consuming, and is regarded as unreliable^34^. Consequently, such experiments do not yield a representative combination of experimental parameters. Response Surface Methodology (RSM) experimental designs, on the other hand, offer an opportunity to take into account the combined effect of several factors, which sheds light on their interactions and the resultant statistical models^35^. Li et al.^36^ demonstrated the potential of RSM to alleviate aflatoxin contamination in maize in their study that used RSM to optimize the plasma treatment conditions for inhibiting *A. flavus *on maize kernels^36^.

The present study investigated the effect of LTP on aflatoxin and fungal load in naturally contaminated maize, identified the optimal decontamination parameters to optimize decontamination to levels below the main international standards, and quantitatively modeled the impact of the variability in the effectiveness of LTP on aflatoxin exposure through maize in endemic regions.

## Materials and methods

### Materials

#### Maize samples

Maize samples (25 g each) were drawn from a blended sample obtained from different parts of Kenya. The samples were naturally contaminated with a fungal load (3–210 cfu/g) and aflatoxin content (66.12–105.98 ppb) before treatment.

#### Microbial analysis

Fungi were enumerated using Rose Bengal Agar with Chloramphenicol (chloramphenicol, 0.1 g/L) (Himedia, Nashik, India). One gram of each milled sample was suspended in 9 mL of diluent and serial dilutions prepared (10^−3^ to 10^−5^). The diluents were plated in triplicates and incubated in parafilm sealed plates for 3–5 days at 26 ± 2^o^C followed by identification using macro and micro-morphological characteristics according to Peterson and Klich^37^.

#### Enzyme immunoassay for total aflatoxin (ELISA)

Aflatoxin analysis of the samples was carried out using the competitive enzyme-linked immunoassay for quantitative detection of aflatoxin B1, B2, G1 and G2 ^1^ (Helica® Total Aflatoxin Assay, Helica Biosystems Inc, United States). The samples were prepared according to the manufacturer’s recommendations. The ground maize samples (5 g) were mixed with 70% methanol at a sample-to-extraction solvent ratio of 1:5 (w/v). The extracted sample was thereafter mixed with 200 µL of HRP-conjugated aflatoxin. Aliquots of 100 µL of each standard and sample was then added to the appropriate mixing well containing the conjugate and mixed three times. An aliquot of 100 µL of the mixture was then transferred to a corresponding antibody coated microtiter well and incubated for 15 min at room temperature. The well contents were thereafter discarded and the micro wells were washed five times by filling each well with phosphate buffered saline Tween wash buffer. Absorbent towels were used to dry the wells (face down) before the introduction of 100 µL of substrate reagent. Finally, 120 µL of stop solution was added to each microwell. The optical density of each microwell was read using a spectrophotometer (model type − 355, Thermo Fisher Scientific, Shanghai, China). The readings were taken using a 450 nm filter. The limit of detection of the kits was 20 ppb. Samples that had more than 20 ppb were further diluted with 70% methanol and confirmatory analysis performed to obtain the accurate total aflatoxin level. High Performance Liquid Chromatography (HPLC) was used for confirmatory analysis of aflatoxins B1, B2, G1 and G2. This involved extraction, filtration, clean up, elution and drying. Extraction of the aflatoxins was done by adding 5 g of ground maize sample to 25 mL of 70% methanol and shaking for 2 h followed by filtration using filter paper (Whatman No.1). Cleaning was done by taking 9 mL of the mixture and drying using nitrogen to < 0.5 mL. This was then diluted to 10 mL using phosphate buffer solution (PBS) and 1 mL of this mixture was then passed through immunoaffinity columns placed on vacuum manifold. This was then washed with 2 × 10 mL water. Derivatization was followed by drying the elute using a nitrogen stream. A 200 µL aliquot of Trifluoroacetic acid was then added, vortexed for 1 min and incubated for 30 min away from light. The sample was then filtered using a 0.2 μm membrane filter (GHP) before injection into the HPLC machine (NEXERA UHPLC SHIMADZU – JAPAN). The type of column used for the analysis was Nova-pak C18 4 μm x 150 mm (WATER CORP – IRELAND). The following operating conditions were set during the process: run time – 30 min; injection volume − 10 µl; column temperature – 35^o^C, velocity – 1.0 mL/minute. Aflatoxins were analyzed as their Trifluoroacetic acid derivatives (TFA) and identified according to their retention times. Quantification was done by using external standard curves.

#### Plasma experiments

Maize samples were exposed to plasma using the plasma unit (Diener electronic GmbH + Co.KG, Ebhausen, Germany) in 100 mm Petri dishes (100 mm diameter) at runs varying in time, pressure, and ionization density as determined in the optimization steps below.

The optimization process using Response Surface Methodology (RSM) largely involves three distinct steps: Generation of statistically designed experimental designs, co-efficient estimation using mathematical modelling, and lastly, response prediction and testing of the significance of the model to the experiment^38^. The experimental design was generated using the Box Behnken Design (BBD) RSM models using the Design Expert 11 software (StatEase, 2020). The optimization formula used was as shown in Eq. 1 ^39^. The parameters considered for optimization were a reduction in the fungal load and a percent reduction in the aflatoxin content. The variable factors were time, pressure and ionization power.

1 $$y = f(x_1, x_2,x_3)$$

Where ***y*** represents the response variables; either percent reduction in the aflatoxin content or fungal load, whilst ***x***_***(1−3****)*_ represents the independent variables time, pressure and ionization power. Their maximum and minimum values (Table 1) were chosen based on similar studies carried out by^32,40–42^.

**Table 1:** Minimum and maximum values of factors selected in the box Behnken design.

Factor	Units	Minimum	Maximum
Time	Seconds	5	1,800
Pressure	pascal	0.1	1.7
Ionization power	Watts	60	200

The percent reduction in the aflatoxin content and fungal load was derived using Eq. 2 according to Behera et al.^39^.

2 $$N = 3^n +3n+n_c$$

Where ***N*** represents the total number of experimental runs, ***n*** is the number of factors and ***n***_***c***_ is the resultant total number of central points. The total percent reduction for aflatoxin and fungal load was calculated according to the Eqs. 3 and 4 respectively.

3 $$Aflatoxin\: percent\: reduction\: = \frac{Initial\: aflatoxin\: level\: -  Aflatoxin\: level\:after\: treatment\:}{Initial\: aflatoxin\: level\:}\times100$$

*Initial aflatoxin level*.

4 $$Fungal\: load\: percent\: reduction\: = \frac{Initial\: fungal\: load - Fungal\: load\: after\: treatment}{Initial\: fungal\: load}\: \:\times \:100$$

#### Statistical analysis

To study the combined effect of the independent factors as well as their interactions with process variables and responses, the data from the decontamination experiment was subjected to generalized regression models and Analysis of Variance (ANOVA) to select the best model and evaluate their coefficients. This was done using the BBD RSM in the Design Expert software (StatEase, 2020). The best fitting model was selected between cubic, quadratic, two factor interaction and linear models fit using Type I sequential sum of squares. Regression analysis and plots of responses and contour plots were also made at the optimized conditions. The F-test was conducted to deduce the statistical significance of the factors. The coefficient of determination (R^2^) was used to assess the amount of variation accounted for by the models and the level of statistical significance of the factors was evaluated at an alpha level of 0.05. Numerical optimization was performed using the Design Expert software (StatEase, 2020) by setting the constraints on the LTP parameters to obtain the values that maximize percent reduction in aflatoxins and fungal load in maize.

#### Quantitative exposure assessment

Aflatoxin exposure was quantitatively modeled in R software for statistical computing using Monte Carlo simulation to take into account variability in exposure, as shown in Table 2.

**Table 2:** Summary of the exposure model: variables, equations, or distribution of the input parameters and data sources.

Process	Variable	Description	Distribution/ Equation	Data Source/reference
Distribution in Aflatoxin Exposure (ng/Kg Body Weight/day)	*C*_*afla*_	Distribution of Aflatoxin Concentration (ng/g) in Food	*trnorm*($\:{\mu\:}_{conc},{\sigma\:}_{conc}$) Where: *trnorm* = truncated normal distribution with lower cutoff = 0 $\:{\mu\:}_{conc}$= mean aflatoxin concentration in maize, $\:{\sigma\:}_{conc}$= standard deviation in aflatoxin concentration in maize	This study
	*B*_*wt*_	Distribution of weight body (Kg)	*trnorm*($\:{\mu\:}_{Bwt},{\sigma\:}_{Bwt}$) Where: *trnorm* = truncated normal distribution with lower cutoff = 0 $\:{\mu\:}_{Bwt}$= mean body weight, $\:{\sigma\:}_{Bwt}$= standard deviation body weight Where distribution lower cutoff = 0	^31^
	*Cons*	Distribution of daily maize intake (g)	*discrete*($\:{P}_{cons},{Q}_{cons\_i}$) Where: *discrete* = discrete distribution $\:{P}_{cons}$= proportion of consumers, $\:{Q}_{cons\_i}$= quantity consumed by each $\:{P}_{cons}$ in meal *i* where $\:i$ is either breakfast, lunch or dinner $\:{Q}_{cons\_i}\:\sim\:uniform\left(mi{n}_{consume{d}_{i}},ma{x}_{consume{d}_{i}}\right)$	^31^
	*E*_*i*_	Aflatoxin Exposure (ng/Kg Body Weight (bw)/day)	$\:\sum\:\left(\frac{{C}_{afla}\times\:Cons}{{B}_{wt}}\right)$ Summing over contribution from consumption during breakfast, lunch and dinner	Calculated
Reduction after plasma treatment	$\:{C}_{red\_i}$	Concentration in of aflatoxin in maize after plasma treatment	*C*_*i*_ = *C*_*i*−1_$\:\times\:$ (1-*d*_*i*_) where $\:{d}_{i}$ is the fraction of the aflatoxin removed due to combination $\:i$ of plasma treatment.	

A truncated-normal distribution was used to model the mean body weight (kg) and concentrations of aflatoxins in maize kernel and maize meal (ng g^–1^). Different proportions of consumers consume various ranges of maize products consumed per meal. Consumption data was therefore modeled using discrete distribution to account for variations in proportion of consumers and a uniform distribution to model the minimum and maximum amounts consumed per meal. The distributions were fitted and 100, 000 Monte Carlo simulations were conducted using *R* software for statistical computing. Sensitivity analysis was conducted by calculating Spearman’s rank correlation between the estimated exposure and the model inputs including aflatoxin concentration, consumption and variation in body weight. Scenario analysis was conducted by modeling the effect of different plasma combinations on exposure. Aflatoxin concentration levels in maize were reduced according to four levels computed from the percent reduction. The levels were computed by splitting the ordered data values into bins that minimize the variance around the expected bin size using *smart_cut* function from *cutr* package in *R* software for statistical computing. To evaluate the effect of the four factor combinations on aflatoxin reduction, Principal Component Analysis (PCA) was performed to identify patterns in the factor combinations that explain the variance in aflatoxin reduction.

## Results

### Aflatoxin and fungi reduction in experimental runs

The RSM methodology modelled an experimental design with time, pressure and ionization power as the independent variables. Using the RSM, seventeen experimental runs were required (Table 3), and the factor combinations yielded the percent reduction in the fungal load and aflatoxin content, as shown in Table 1.

Seventeen experiments were required for the response surface methodology based on the Box-Behnken design. Based on the experimental design, the factor combinations yielded different responses as presented.

**Table 3:** Box Behnken design for optimization of the decontamination process and the observed reduction in fungal load and aflatoxin levels.

Run	Predictor variables			Response variables	
	Time (Sec)	Pressure (pascal)	Ionization power (Watts)	% reduction in fungal load	% reduction in aflatoxin level
1	902.5	0.9	130	19.4	70.2
2	902.5	0.9	130	20.0	73.8
3	902.5	1.7	60	63.6	73.6
4	902.5	0.1	60	55.6	74.9
5	902.5	1.7	200	58.3	75.7
6	1800	0.9	60	83.3	82.5
7	902.5	0.1	200	87.5	82.6
8	1800	1.7	130	58.3	80.9
9	1800	0.1	130	93.3	77.8
10	5	1.7	130	27.3	64.4
11	902.5	0.9	130	66.7	73.3
12	5	0.9	200	20.0	68.6
13	902.5	0.9	130	55.6	75.2
14	5	0.9	60	8.6	66.3
15	5	0.1	130	45.5	63.7
16	902.5	0.9	130	35.3	71.2
17	1800	0.9	200	100.0	80.1

### Optimal aflatoxin reduction parameters using RSM modelling

The best fitting model was the linear model (F-values = 46.13, sequential p-value = 0.0001) with a non-significant lack of fit test (F-value = 2.88, *p* = 0.16) (Table 4). The model accounted for a high amount of variation (adjusted R^2^= 0.74). There was a reasonable agreement between the observed and predicted reduction of aflatoxins by the models with the predicted R^2^(0.59) and the adjusted R^2^ having a difference of less than 0.2.

An ANOVA on individual and combined effects of factors (time, pressure, and ionization power) on the percent reduction in the aflatoxin content indicated that only the impact of time was significantly different (*p* < 0.0001) (Table 4).

**Table 4:** Analysis of variance for linear model prediction for reduction in aflatoxin content.

Source	Sum of Squares	df	Mean Square	F-value	*p*-value
**Model**	440.61	3	146.87	15.89	0.0001^a^
A-Time	426.36	1	426.36	46.13	< 0.0001
B-Pressure	2.56	1	2.56	0.2772	0.6074
C-Ionization power	11.68	1	11.68	1.26	0.2813
**Residual**	120.15	13	9.24		
Lack of Fit	104.10	9	11.57	2.88	0.1602^b^
Pure Error	16.05	4	4.01		
**Cor Total**	560.76	16			

^a^Significant model fit; ^b^Non-significant lack of fit text

**Fig. 1:**
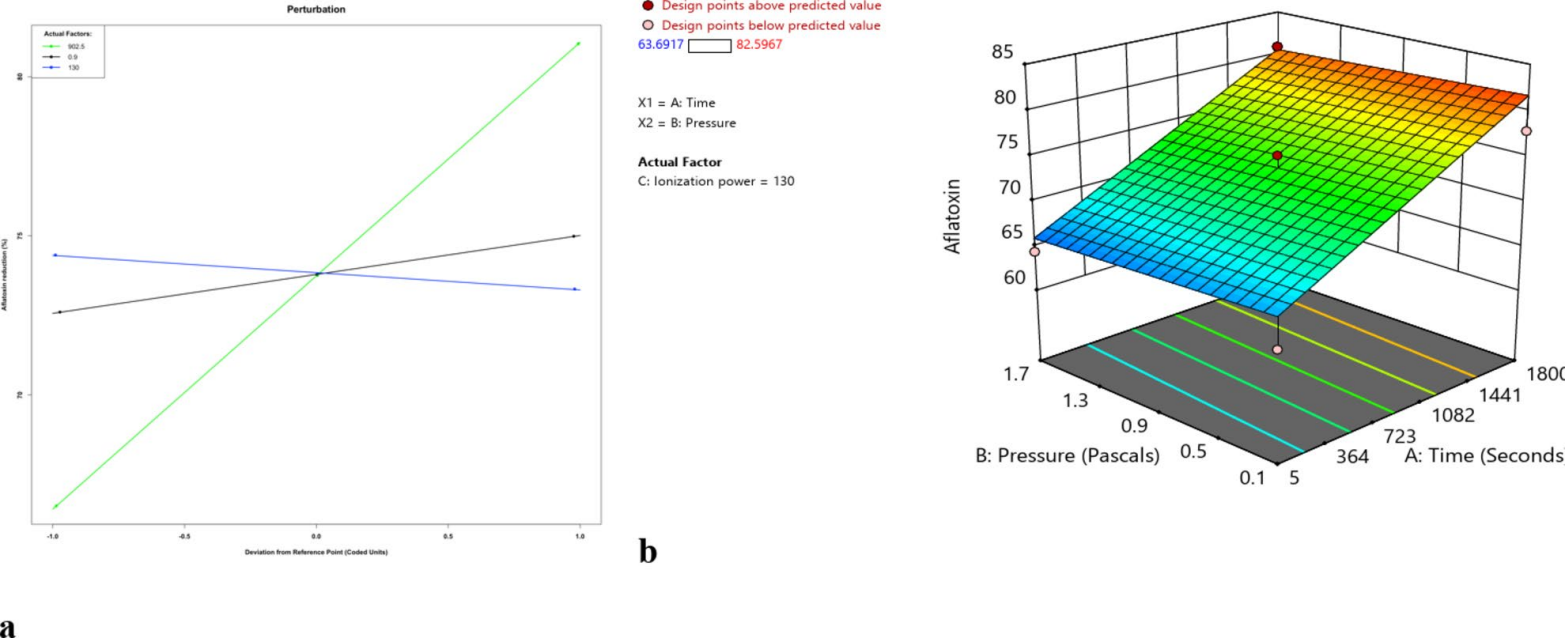
(**a**) Perturbation plot showing the main effect of individual factors (time, pressure and ionization power) on reduction in aflatoxin (A = time in seconds, B = pressure in pascal, C = power in watts) and (**b**) combined effect of pressure and time on percent reduction in aflatoxin content.

 The % reduction in aflatoxin content increased with increasing exposure time and ionization power (Fig. 1, a). There was, however, a negative relationship between pressure and aflatoxin reduction but this effect did not significantly interact with exposure time (Fig. 1, b).

Equation 5 shows the effect of the predictors on the reduction of aflatoxin in maize.

5 $$Aflatoxin\: (Percent\: reduction) \:=\: 73.81 + 7.30\: Time\: -\: 0.57\: Pressure + 1.21\: Ionization\: Power$$

There was a 7.3 times increase in aflatoxin percent reduction for every unit increase in exposure time when pressure and ionization power are held constant.

### Optimal fungal load reduction parameters using RSM modelling

The linear model fitted best with a significant fit compared to cubic and quadratic models (F-value = 7.22; sequential *P* = 0.0043), a non-significant lack of fit test (F-value = 0.7273; *p* = 0.68) and accounted for a considerable amount of variation (adjusted R^2^ = 0.54) (Table 5). There was a reasonable agreement between the observed and predicted reduction in fungal load by the models with the predicted R^2^(0.42) and the adjusted R^2^ having a difference of less than 0.2.

An ANOVA on individual and combined effects of factors (time, pressure, and ionization power) on the percent reduction in the fungal content indicated that only the impact of time was significantly different (*p* = 0.0008) (Table 5).

**Table 5:** Analysis of variance (ANOVA) for linear model prediction of percent reduction in fungal load.

Source	Sum of Squares	df	Mean Square	F-value	*p*-value
**Model**	7891.01	3	2630.34	7.22	0.0043^a^
A-Time	6827.04	1	6827.04	18.74	0.0008
B-Pressure	689.46	1	689.46	1.89	0.1921
C-Ionization power	374.51	1	374.51	1.03	0.3291
**Residual**	4734.75	13	364.21		
Lack of Fit	2938.84	9	326.54	0.7273	0.6835^b^
Pure Error	1795.92	4	448.98		
**Cor Total**	12625.76	16			

^a^Significant model fit; ^b^Non-significant lack of fit text

**Fig. 2:**
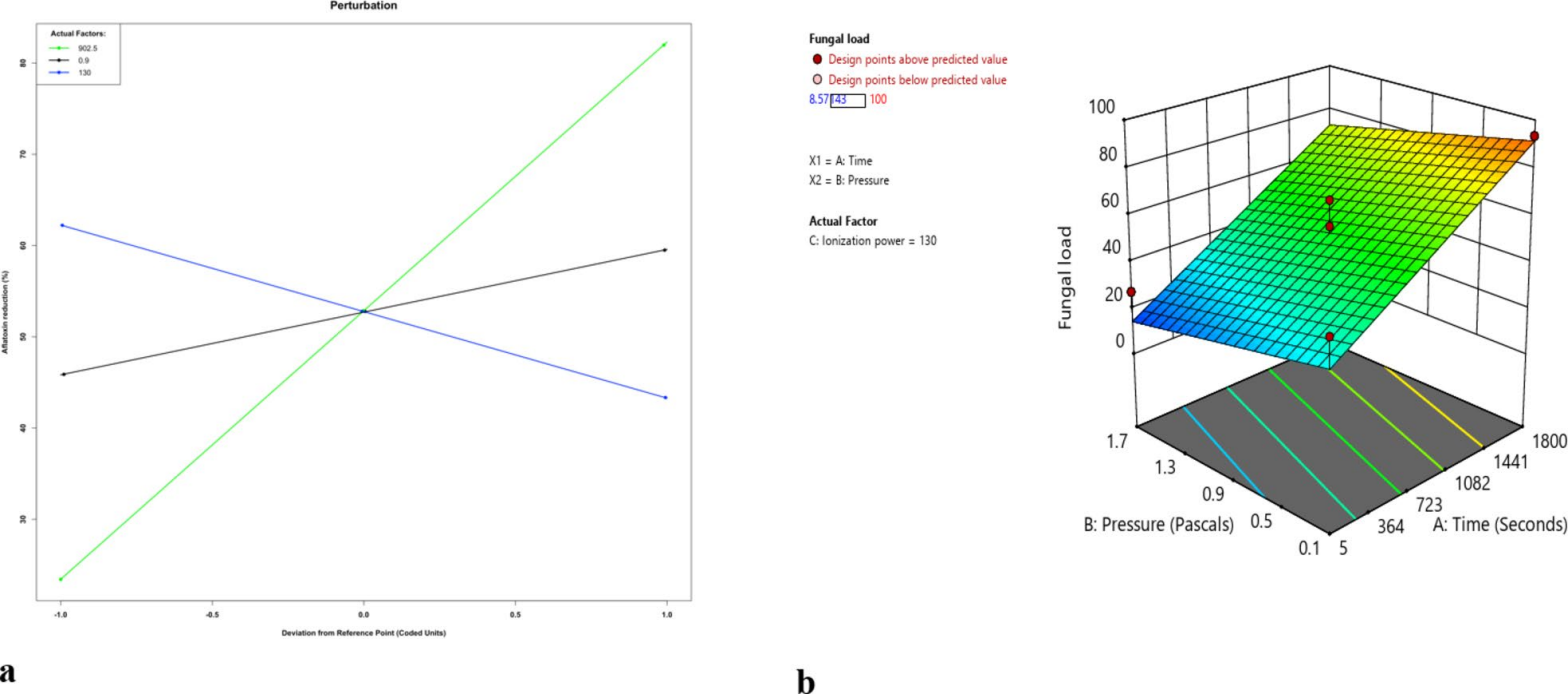
(**a**) Perturbation plot showing the main effect of individual factors (time, pressure and ionization power) on reduction in fungal load (A = time in seconds, B = pressure in pascal, C = power in watts) and (**b**) combined effect of pressure and time on percent reduction in fungal load.

 The % reduction in fungal content increased with increasing exposure time and ionization power (Fig. 2, a). There was, however, a negative relationship between pressure and fungal content reduction but this effect did not significantly interact with exposure time (Fig. 2, b).

Equation 6 shows the effect of the predictors on the reduction of aflatoxin in maize.

6 $$Fungal\: load\: (Percent\: reduction)\: = \:52.84 + 29.21\: Time - 9.28\: Pressure + 6.84\: Ionization\: Power$$

There is 29.2 times increase in fungal content reduction for every unit increase in exposure time with pressure and ionization power remaining constant.

### Optimal factors generated by the response surface methodology

**Fig. 3:**
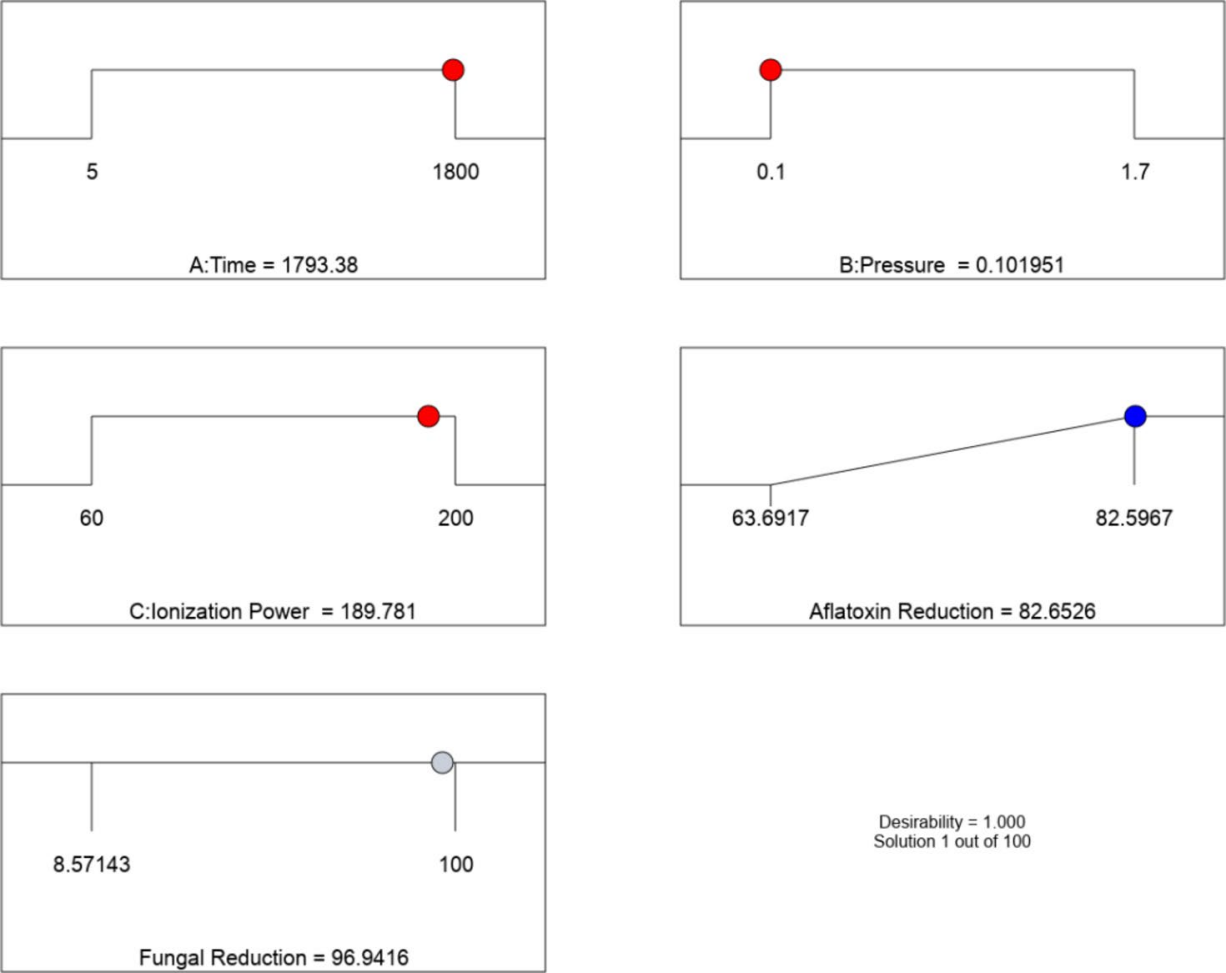
Optimal points for aflatoxin and fungal load reduction as generated using the RSM methodology.

The optimal points for each factor obtained by setting the constraints on the LTP parameters to obtain the values that maximize percent reduction in aflatoxins and fungal load in maize are shown in Fig. 3. The optimal parameters were a time of 1793.4s, the pressure of 0.1 pascal, and ionization power of 189.8 watts. In turn, the program also suggested the resultant reduction levels for each response variable. The optimal percent reduction in the aflatoxin content using these parameters was deduced to be 82.6% (standard error = 1.9), and the percent reduction of fungal load was 96.9% (standard error = 11.8).

**Fig. 4:**
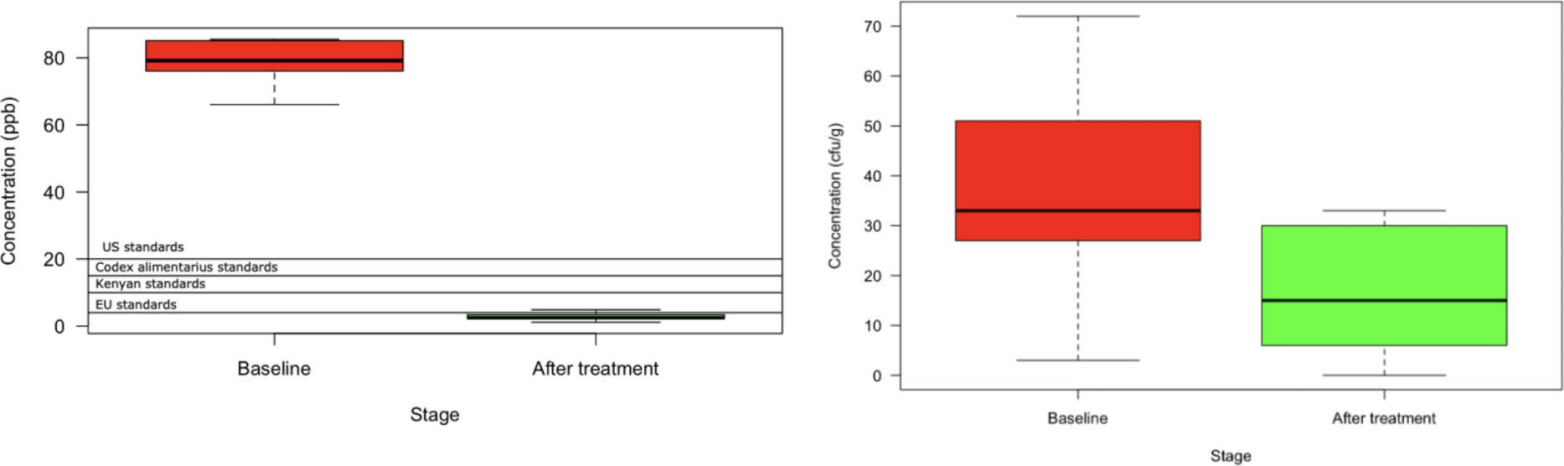
Aflatoxin and fungal concentrations in maize kernels before and after plasma treatment.

The median aflatoxin contamination levels in maize kernels before plasma treatment was 79.18 (interquartile range (IQR): 76.1–82.4) µg kg^–1^while the concentrations fell to a median of 2.5 (IQR: 2.2–3.4) µg kg^–1^ (Fig. 4) after treatment. None of the samples met selected local and main regional international limits before treatment while the median concentrations after treatment were all below Kenyan (10 µg kg^–1^), USA (20 µg kg^–1^), European Union (4 µg kg^–1^) and Codex Alimentarius Commission limits (15 µg kg^–1^) (Fig. 4). There was a corresponding drop in fungal concentration after plasma treatment from a median of 33 (IQR: 17–51) cfu/g before treatment to 32.1 (IQR: 6–30) cfu/g after treatment (Fig. 4).

### Dietary exposure assessment

**Fig. 5:**
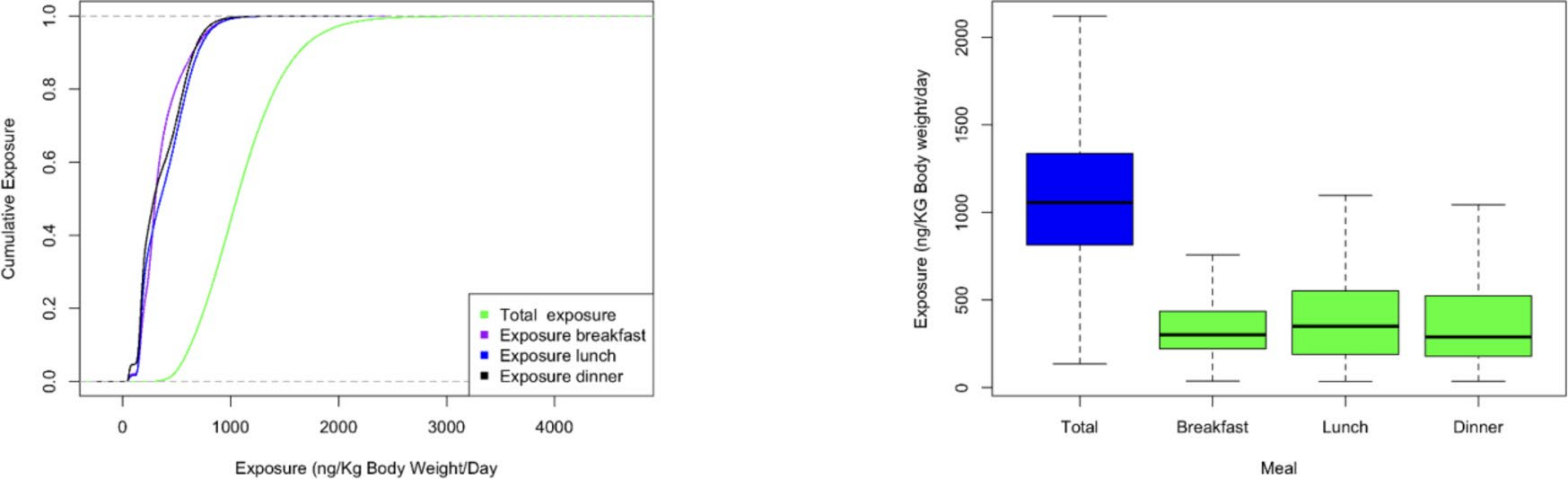
Cumulative probability distribution and distribution of exposure to aflatoxin from maize presented as total daily aflatoxins and the contribution of consumption during breakfast, lunch and dinner to this exposure.

Figure 5shows the daily cumulative probability distribution and comparison of dietary exposure to aflatoxins from maize. There was a median daily dietary exposure to aflatoxins of 1056.7 (IQR: 814.4–1337.5) ng kg^–1^bw day^–1^. A comparison of the exposure between the meals indicated a slight but negligible increase in exposure between breakfast (median: 301.7; IQR: 221.2–435.8), lunch (median: 350.3; IQR: 189.9–553.2) and dinner (median: 357; IQR: 178.1–524.7) ng kg^–1^bw day^–1^.

### Sensitivity analysis

**Fig. 6:**
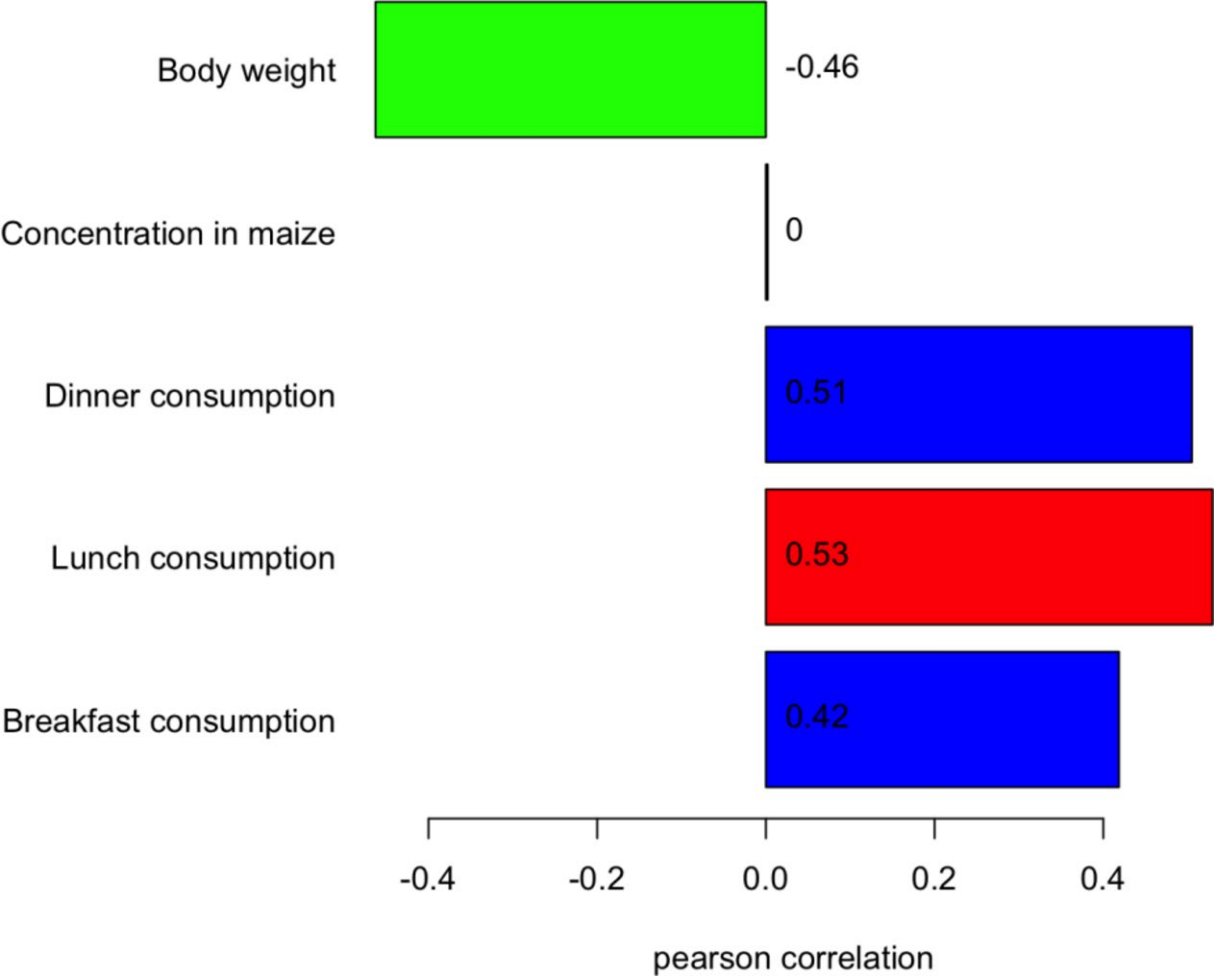
Tornado chart depicting sensitivity analysis results on important factors affecting daily exposure to aflatoxins. Values adjacent or inside the bars indicate the Spearman’s correlation coefficient explaining the magnitude each of the model inputs has on exposure to aflatoxins.

The relative contribution of model inputs on aflatoxin exposure was determined using Spearman’s rank correlation and presented in the tornado diagram (Fig. 6).

The quantity consumed during lunch (Spearman’s correlation coefficient (ρ) = 0.53) had the highest effect on the variability in consumer exposure to aflatoxins (Fig. 6) followed by consumption during dinner (ρ = 0.51). Body weight was negatively related to exposure (ρ = −0.46) indicating higher exposure to individuals with lower body weight. The aflatoxin concentration in maize ((ρ = 0.003) had the least influence on the variability in consumer exposure to aflatoxins (Fig. 6).

### Decrease in aflatoxin concentrations from plasma treatment

**Fig. 7:**
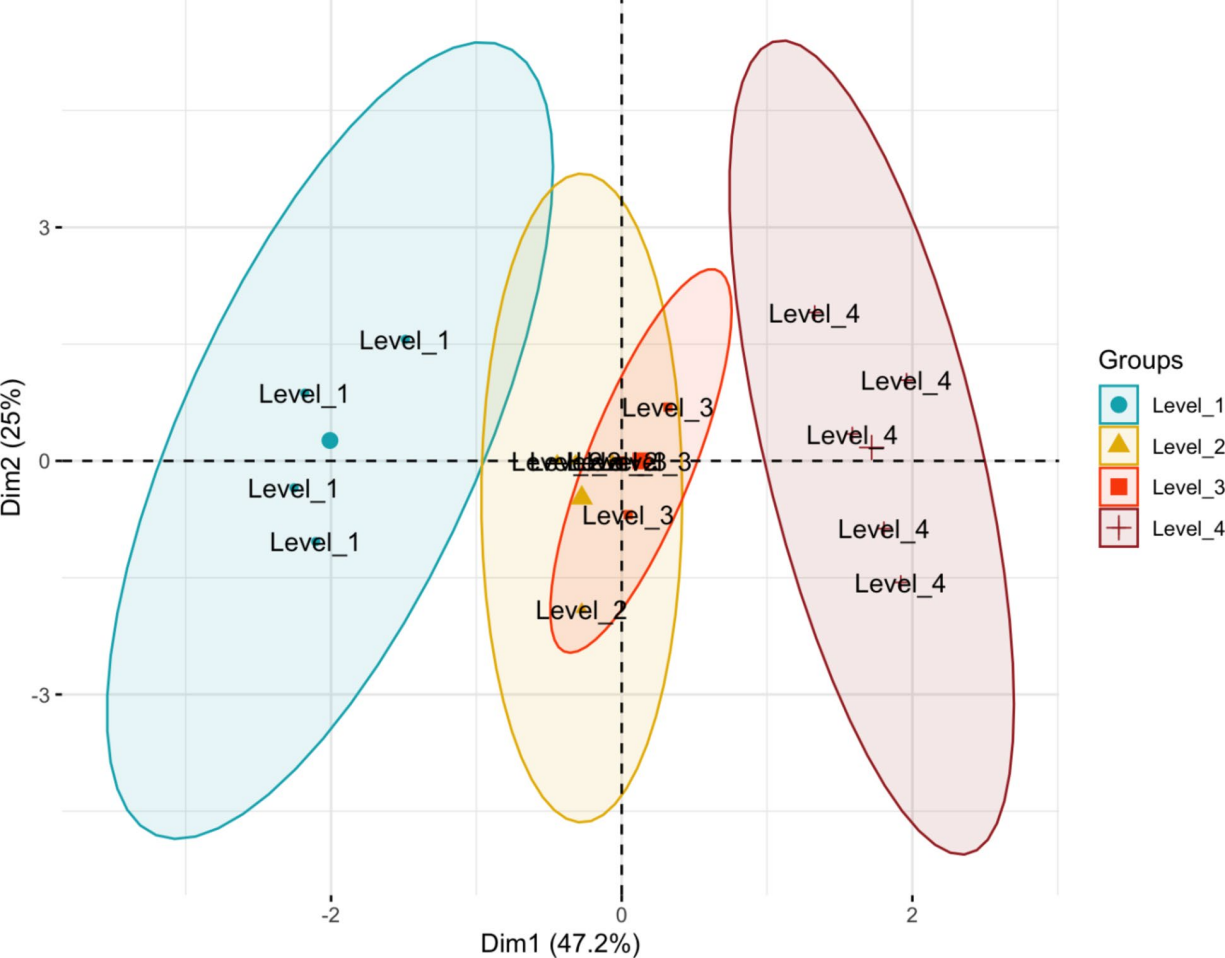
Principal component analysis (PCA) of the factor combinations and percent aflatoxin reduction labelled by ordinal reduction levels.

The categorized reduction levels in aflatoxin concentration using plasma were used to calculate the possible aflatoxin exposure levels upon treatment of maize. The baseline distributions of concentrations from untreated maize were reduced at an average of the levels, namely, [63.69,70.2) (combination 1), [70.2,73.8) (combination 2), [73.8,77.82) (combination 3) and [77.82,82.6] (combination 4). The exposure estimates were computed by repeating the simulations after each reduction level and recording the new exposure estimates (Fig. 7). PCA of the factor combinations and percent aflatoxin reduction indicated that the first two axes (PCA axis 1 and) of the PCA explain 72.2% of the variance. The PCA with these combination labels indicated a separation of the first and fourth combination while there was a considerable overlap between the second and third combinations. The findings from the PCA indicate that the first and fourth factor combinations have distinct effects on aflatoxin reduction, while the second and third combinations perform similarly. This supports the findings above that specific combinations of factors may be more effective at reducing aflatoxin contamination, providing insights into potential optimization strategies for food safety interventions. The overlap in PCA between the second and third combinations implies that altering the factors in these combinations might not yield significantly higher decontamination.

**Fig. 8:**
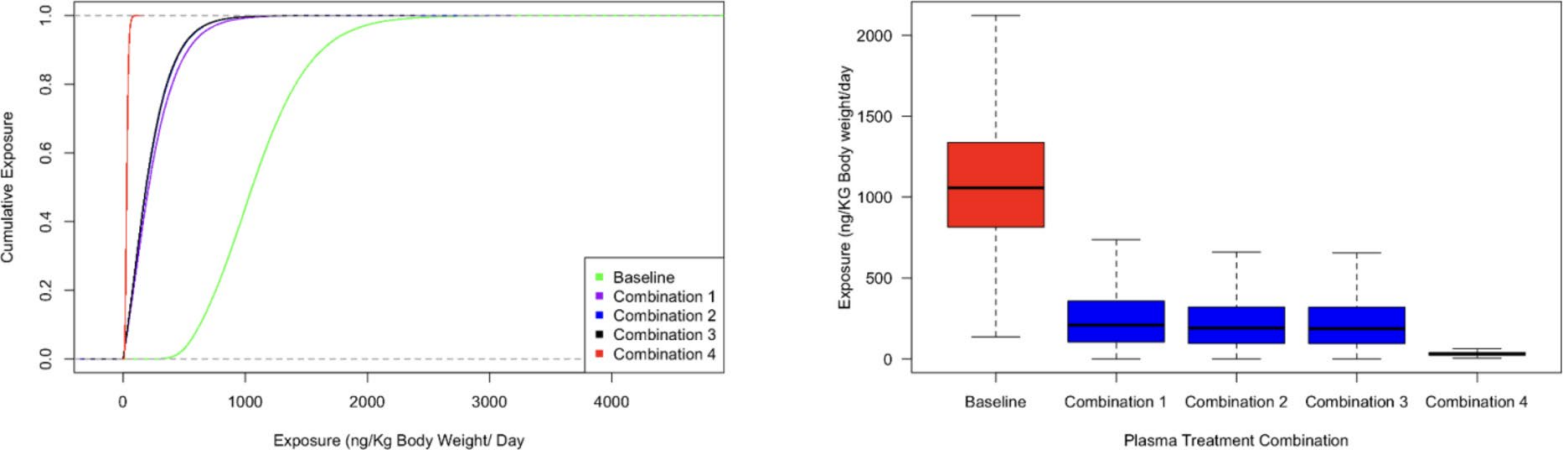
Reduction in aflatoxin exposure by plasma treatment for ordinal categories of percent reduction in aflatoxin concentrations using plasma in maize.

The median aflatoxin exposure in maize kernels would be reduced from 1056.7 (IQR: 814.4–1337.5) ng kg^–1^in the baseline scenario to 209.4 (IQR: 104.3–357.2) ng kg^–1^, 190.1 (IQR: 95.6–321.4) ng kg^–1^, 187.2 (IQR: 94.3–318.6) ng kg^–1^, 29.9 (IQR: 22.5–38.7) ng kg^–1^ for treatment combinations 1, 2, 3 and 4 respectively (Fig. 8).

As supported by the PCA (Fig. 7), the second and third combinations implies that altering of the factors in the second and third combinations did not yield significant changes in exposure.

## Discussion

We optimized the application of the LTP and its parameters in reducing aflatoxin contamination in maize to below set limits for human consumption. We further modeled the reduction in consumer exposure to aflatoxins through consuming maize in endemic regions.

The prediction of the percent reduction in fungal load using the linear model generated by RSM methodology revealed that time but not pressure and ionization power significantly influenced the percent reduction in aflatoxin and fungal contamination in maize. An increase in the time and ionization power pressure leads to a corresponding decrease in the percent fungal load and aflatoxin contamination while pressure inversely but non significantly influenced fungal load and aflatoxin contamination.

The percent reduction in fungal load was 96.9% (standard error = 11.8) when the optimal parameters of 1793.4s, pressure of 0.1 pascal and ionization power of 189.8 watts were used. A study on the effect of LTP on fungi in laboratory-contaminated maize^43^ demonstrated the potential to inactivate fungi in maize by 33.33% at a higher plasma parameter of 360 W power at a similar exposure time of 1800s to that of our study. The variation in application pressure was not included in the study by Silva et al.^43^ though longer exposure time was similarly associated with increased LTP potency. A fungal inactivation optimization study in laboratory-contaminated maize samples reported a 79% reduction in *A. flavus *spores contamination at the optimized plasma treatment parameters consisting of 90 Pa plasma degree, treating time of 90 s, and power of 500 W^36^. The mechanism of action of LTP on fungi is linked to the reactive species (oxygen) in the plasma. These are thought to interfere with several pathways of the fungal structure leading to its destruction. Some of the functions curtailed by LTP plasma are inhibition of the cell membrane function, apoptosis^44^, intracellular nanostructural changes, morphological changes in cell membrane and increased permeability^42^and finally through oxidation of intracellular organelles^45,46^. The role of reactive nitrogen species (RNS) and UV light has not been exhaustively researched and further research is needed in order to understand their role in fungal inactivation^18^. LTP is a promising technology due to the persistence of the fungistatic effect. For instance, Silva et al.^47^ reported no growth of *Aspergillus flavus* after 6 days of incubation of samples treated for 15–20 min and slow growth for the samples treated for 2–12 min as compared to the control sample. The plasma conditions were, however, not specified in their study.

Similarly, to the reduction in fungal load, exposure time was an important parameter in aflatoxin decontamination in maize. Change in the pressure and ionization power did not also have any significant effect on the aflatoxin level. The percent reduction in the aflatoxin content of 82.6% (standard error = 1.9) at the optimal LTP of 1793.4s, pressure of 0.1 pascal and ionization power of 189.8 watts indicates the potential of LTP to effectively reduce aflatoxin concentration to below major international limits in naturally contaminated maize. Whereas all samples exceeded the local, main regional and codex international limits before treatment, the median concentrations after LTP treatment were all below Kenyan (10 µg kg^–1^), USA (20 µg kg^–1^), European Union (4 µg kg^–1^) and Codex Alimentarius Commission limits (15 µg kg^–1^). These example limits cover the range in maximum tolerable level of total aflatoxins in maize and peanuts globally which ranges from 4 to 20 µg kg^[–1 48^. This supports the LTP as a potential approach in both reducing the public health burden of aflatoxins and facilitating international trade. Our results indicate that optimizing the LTP parameters using RSM was effective in meeting the goals of a reduction in aflatoxin that is comparable to that obtained in laboratory challenge tests exploring the potential of plasma technology in destroying aflatoxins in maize. This is despite the potentially high variation in concentration, matrix effect and distribution of aflatoxins in the matrix for naturally contaminated maize that may attenuate the degradation of aflatoxins by plasma technologies. For instance, Ten Bosch et al.^30^reported that pure mycotoxins were rapidly degraded completely after 60 s of treatment while these rates were reduced to varying extents when mycotoxins were introduced on rice. They also reported that the degradation of mycotoxins is dependent on the matrix and their structure with pure toxins being degraded faster than a mixture of several mycotoxins^30^. Shi et al.^32^ reported a 62 and 82% decrease in aflatoxin content in laboratory contaminated maize after 1 and 10 min of LTP treatment using an ionization power of 200 W which is close to the optimum value of 189 W in the present study. Wielogorska et al.^33^ reported a reduction by between 65% and 64% of aflatoxin B_1_ and fumonisin B_1_ respectively in Maize after 10 min of plasma exposure.

The efficacy of plasma on aflatoxin degradation is influenced by three agents: heat, UV radiation and the reactive species produced during ionization of the reactive gas^49^. The conditions in heat (< 60^o^C) and UV radiation (50 µW/cm^−2^) do not meet the threshold for degradation of the mycotoxins. To degrade mycotoxins higher UV intensities are required of as high as 800 µW/cm^[−2 50^. Thus, in the present case, the degradation may be attributed to the action of the reactive species on the functional groups, different active rings, and the double and triple bonds in the structure of the mycotoxins. This, in turn, leads to the production of lesser toxic compounds than the original mycotoxin. An increase in the power, pressure, and exposure time suggests higher production of reactive species and, thus, higher efficacy of the generated LTP^49^. Our study indicates that among these variables, exposure time may be a more important consideration for optimizing the efficacy of LTP on aflatoxin degradation.

The quantitative exposure assessment indicated a median daily dietary exposure to aflatoxins of 1056.7 (IQR: 814.4–1337.5) ng kg^–1^ bw day^−1^. The range of these exposure estimates (174.1- 5018.6 ng kg^–1^ bw day^−1^) overlaps with the estimates from the exposure assessment by Kilonzo et al.^31^who reported mean dietary exposure to aflatoxin in maize kernels of 292 ± 1567 ng kg^–1^ bw day^−1^ though the maize in our study was sampled from multiple regions.

A comparison of the average and variability (± representing standard deviation) in reduction of exposure by LTP to 31.7 ± 12.6 ng kg^–1^ bw day^−1^ (combination 4) from this study with that from existing technologically implemented approaches indicates that the optimized reduction in our study would be comparable but more precise as illustrated by the lower variability. Kilonzo et al.^31^ reported an average and variability in reduced exposure of 171 ± 1035 by cleaning, 19 ± 111 by treatment with ammonium persulfate, 59 ± 62 by milling and 27 ± 154 ng kg − 1 using traditional dehulling (muthokoi). LTP is also advantageous in that decontamination occurs at ambient temperature and pressure without additives which achieves decontamination without any detectable changes in quality.

Sensitivity assessment indicated that higher aflatoxin exposure estimates were most correlated with higher consumption and lower body weight. This raises a concern about the impact of aflatoxin exposure on children in lower body weight ranges. Previous evidence pooled using a meta-analysis indicated that aflatoxin exposure was associated with an enhanced risk of growth impairment in infants^51^. Though lower consumption would be associated with lower aflatoxin exposure, control measures need to be tailored towards reducing concentrations in maize given that maize constitutes a major source of both animal feed ingredients globally and a calorie intake in many countries, for instance, up to 70% in Kenya^52^. This lays an emphasis on the importance of LTP in effectively reducing the public health and economic burden resulting from aflatoxin exposure or food waste due to concentrations in grain exceeding the set limits.

## Conclusion

From the results, it can be concluded that LTP, when optimized using RSM, does have efficacy in reducing the fungal load and aflatoxin in naturally contaminated maize from an endemic region to achieve levels in compliance with the set limits. During plasma treatment, an increase in exposure time led to a significant decrease in both fungal load and aflatoxin content. To achieve an optimal reduction of aflatoxin content of 82.6% and a fungal load of 96.9%, an exposure time of 1793.4s sec, a pressure of 0.98 pascal, and an ionization power of 189.8 W is required. Despite the variation in aflatoxin content in naturally contaminated maize, the best LTP combination reduced exposure to aflatoxin comparably but more precisely with lower variability than existing technological interventions. Future optimization studies are needed incorporating other factors such as temperature and upscaling aspects such as volume as independent factors to refine the optimization of the decontamination process considering additional outcomes such as organoleptic, physical and chemical changes in the food matrices after treatment. Up-scale and pilot studies are needed to assess the practical feasibility of the technology while applying implementation research or science to perfect maize detoxification parameters.

## Data Availability

The datasets used and/or analyzed during the current study are available from the corresponding author on reasonable request.
